# Polyaniline-intercalated manganese dioxide nanolayers as a high-performance cathode material for an aqueous zinc-ion battery

**DOI:** 10.1038/s41467-018-04949-4

**Published:** 2018-07-25

**Authors:** Jianhang Huang, Zhuo Wang, Mengyan Hou, Xiaoli Dong, Yao Liu, Yonggang Wang, Yongyao Xia

**Affiliations:** 1 Department of Chemistry and Shanghai Key Laboratory of Molecular Catalysis and Innovative Materials, Institute of New Energy, iChEM (Collaborative Innovation Center of Chemistry for Energy Materials) Fudan University, 200433 Shanghai, China; 2 School of Materials Science and Engineering, Nanchang Hangkong University, 330063 Nanchang, China

## Abstract

Rechargeable zinc–manganese dioxide batteries that use mild aqueous electrolytes are attracting extensive attention due to high energy density and environmental friendliness. Unfortunately, manganese dioxide suffers from substantial phase changes (e.g., from initial α-, β-, or γ-phase to a layered structure and subsequent structural collapse) during cycling, leading to very poor stability at high charge/discharge depth. Herein, cyclability is improved by the design of a polyaniline-intercalated layered manganese dioxide, in which the polymer-strengthened layered structure and nanoscale size of manganese dioxide serves to eliminate phase changes and facilitate charge storage. Accordingly, an unprecedented stability of 200 cycles with at a high capacity of 280 mA h g^−1^ (i.e., 90% utilization of the theoretical capacity of manganese dioxide) is achieved, as well as a long-term stability of 5000 cycles at a  utilization of 40%. The encouraging performance sheds light on the design of advanced cathodes for aqueous zinc-ion batteries.

## Abstract (web summary)

Zn-MnO_2_ batteries offer high energy density, but phase changes that lead to poor cathode stability hinder development of rechargeable versions. Here the authors report structurally reinforced polyaniline-intercalated MnO_2_ nanolayers that boost performance by eliminating phase transformation.

## Introduction

In light of pressing concerns regarding environmental pollution and climatic deterioration associated with the combustion of fossil fuels, building a low-carbon society that is based on renewable energy sources has gained widespread attention. However, the utilization of renewable energy sources such as wind and solar requires a safe, green, economic, and efficient electrochemical energy conversion system that can accommodate/smoothen the intermittency of renewable power^1–4^. As a result, aqueous Li^+^ (or Na^+^) batteries are attracting extensive attention due to safety and environmentally friendliness that arise from the use of mild aqueous electrolytes containing Li^+^ (or Na^+^)^5–16^. Unfortunately, electrode materials for Li^+^ (or Na^+^) storage in aqueous electrolytes generally suffer from low capacity (<150 mA h g^−1^)^5–16^, which should be remedied with large-scale energy storage. In such situations, the electrode materials for Zn^2+^ storage in mild aqueous electrolytes have entered researchers’ spotlight. For example, copper hexacyanoferrate^17,18^, V_2_O_5_^3,19,20^, and MnO_2_^21–25^ have been recently reported for Zn^2+^ storage. Among these materials, MnO_2_ attracts much attention because of its high theoretical capacity (308 mA h g^–1^), low cost, and low toxicity^23–25^. As the most widespread primary battery, Zn–MnO_2_ alkaline battery has been commercialized for a very long time. However, the development of rechargeable Zn–MnO_2_ battery was dramatically hindered by the poor reversibility of MnO_2_ in alkaline electrolyte^26,27^.

Recently, the reversible Zn^2+^ and/or H^+^ insertion into a MnO_2_ host in a mild aqueous electrolyte was demonstrated^21–25^, triggering enthusiasm for the development of a rechargeable Zn–MnO_2_ battery using a mild aqueous electrolyte. Various manganese dioxide phases, including α-MnO_2_^22,23,28–31^, β-MnO_2_^24^, γ-MnO_2_^32^, δ-MnO_2_^33^, spinel-type MnO_2_^34^, and other types^35–37^, have been reported as host materials for Zn^2+^/H^+^ insertion in a mild aqueous electrolyte. However, no matter what the original architecture is, the MnO_2_ hosts suffer serious structural transformation during cycling processes and transform into layered manganese oxide phases with interlaminar water molecules^24,31,32^ (Supplementary Fig. 1). The formation of the layered structure should be attributable to manganese dissolution and the insertion of hydrated Zn^2+^ (i.e., [Zn(H_2_O)_6_]^2+^) and H^+^ (i.e., H_3_O^+^) (see Supplementary Fig. 1 and Supplementary Note 1). With coordinated water molecules, the strong electrostatic repulsion between Zn^2+^ (or H^+^) and the host material can be diminished effectively^19,31^. That is to say, theoretically, that the layered structure with 1 × ∞ tunnels and extended interlayer spacing are advantageous for the storage of guest-hydrated cations. However, during the phase-change process (i.e., from α-, β-, γ-phase to layered structure with interlaminar water), large volumetric change leads to significant capacity fading^24^. In addition, with the insertion of a large amount of hydrated cations, the layered structure of manganese oxide will collapse during the charge/discharge process^33,35^, which aggravates capacity fading. As a result, when cycled with high charge/discharge depth, the MnO_2_ electrode generally exhibits very poor stability. Up to the present, the stable cycling of a MnO_2_ electrode with the utilization of >90% (~277 mA h g^−1^ = 308 mA h g^−1^ × 90%) has never been reported, to the best of our knowledge. Currently, the best reported cycle life of MnO_2_ in a mild aqueous electrolyte with high utilization of 84% (~260 mA h g^−1^) is 45 cycles, which was achieved by Liu’s group^23^. Very recently, Chen et al. demonstrated an improvement to 150 cycles with a lower utilization of 75% (~230 mA h g^−1^)^24^. Although there are some reports about high stability (more than 5000 cycles) of a MnO_2_ cathode in a mild aqueous electrolyte, excellent stability has been achieved with very low utilization of the theoretical capacity for MnO_2_ (<30%)^23,25^. Therefore, it is still a great challenge to efficiently utilize the high capacity of a MnO_2_ cathode in a mild aqueous electrolyte.

As mentioned above, the capacity fading of a MnO_2_ cathode arises from both the phase transformation and the instability of H_2_O-intercalated layered structure. Directly using the layered MnO_2_ as an electrode material, which can avoid phase transformations while intercalating a guest polymer into MnO_2_, to strengthen the extended layered structure is a promising solution. Here we prepare the polyaniline (PANI)-intercalated MnO_2_ nanolayers through an interface reaction. The nanoscale size of the layered MnO_2_ and the guest polymer in the interlayer efficiently facilitates the charge storage and strengthen the extended layered structure, and thus as-prepared PANI-intercalated MnO_2_ nanolayers exhibit high-rate capability and a long cycling life. Even with a high utilization of 90% (~280 mA h g^−1^), the PANI-intercalated MnO_2_ nanolayers still display a very stable cycling performance, which is superior to previous reports. Furthermore, a detailed investigation is performed to clarify the co-insertion mechanism of Zn^2+^ and H^+^.

## Results

### Structural characterization

**Fig. 1:**
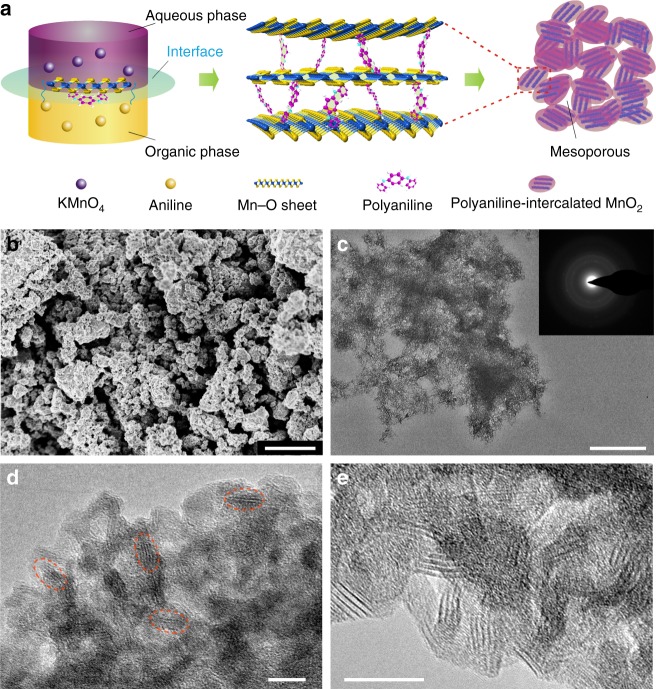
Preparation and characterization of the polyaniline-intercalated MnO_2_ nanolayers. **a** Schematic illustration of expanded intercalated structure of polyaniline (PANI)-intercalated MnO_2_ nanolayers. **b** Scanning electron microscopy image, **c** transmission electron microscopy (TEM) image (the inset shows the corresponding selected-area electron diffraction image), and **d** high-resolution (HR)-TEM image of the PANI-intercalated MnO_2_ nanolayers. The red dashed outlines are used to clarify the morphology profile and particle size of the MnO_2_ nanolayers. **e** HR-TEM image of the PANI-intercalated MnO_2_ nanolayers with heat treatment at 400 °C to remove the shield of PANI. Scale bars, **b** 1 μm; **c** 500 nm, and **d**, **e** 10 nm, respectively

The PANI-intercalated MnO_2_ is prepared by a simple one-step inorganic/organic interface reaction (Fig. 1a), which was developed by our group^38^. At the interface of the organic phase (i.e., CCl_4_-containing aniline monomer) and the inorganic phase (i.e., KMnO_4_ aqueous solution), the chemical oxidation polymerization of aniline and the reduction of MnO_4_^2−^ occur simultaneously, facilitating the layer-by-layer assembly of the layered manganese dioxide and polyaniline (Fig. 1a). Furthermore, the diffusion of aniline from the organic phase to the inorganic phase and the production of PANI restrict the growth of MnO_2_ to two dimensions. Finally, the PANI-intercalated MnO_2_ nanolayers gather together to form a mesoporous structure. Figure 1b presents the scanning electron microscopy (SEM) image of the as-prepared sample, showing a grainy morphology that comprises aggregates of primary particles. Examination of transmission electron microscopy (TEM) data shown in Fig. 1c indicates that the PANI-intercalated MnO_2_ nanolayers composite exhibits a spongiform structure. The diffraction rings obtained from selected-area electron diffraction (SAED) analysis (inset of Fig. 1c) indicate polycrystalline character of the sample. The high-resolution transmission electron microscopy (HR-TEM) image (Fig. 1d) reveals that the PANI-intercalated MnO_2_ nanolayers possess a typical size around 10 nm and a distinct mesoporous structure, and the MnO_2_ nanolayers show an expanded interlayer space (~1.0 nm). The mesoporous structure is further supported by an obvious hysteresis loop in the nitrogen adsorption–desorption isotherms (Supplementary Fig. 2a), which indicate a large surface area of 277 m^2^ g^−1^ and a pore size that is mainly centered at 4 nm (Supplementary Fig. 2b). It should be noted that the layered structure of the MnO_2_ is not very apparent in Fig. 1d because of the shielding of PANI. In order to clarify this point, the PANI-intercalated MnO_2_ composite was heat treated at 400 °C in air for several minutes to obtain a clear view of the intercalated structure (Fig. 1e). After heat treatment to partially remove the shielding of PANI, the expanded interlayer space can be clearly detected in Fig. 1e. Certainly, the result of Fig. 1e also demonstrates that heat treatment at 400 °C did not destroy the expanded layer structure, indicating a good structure stability of the PANI-intercalated MnO_2_ nanolayers. The PANI in the composite was characterized with Fourier transform infrared (FT-IR) spectroscopy (Supplementary Fig. 3), and the weight percentage of PANI (5 wt%) was determined with thermogravimetric (TG) analysis (Supplementary Fig. 4). The broad peaks in the powder X-ray diffraction (XRD) pattern of the PANI-intercalated MnO_2_ nanolayers composite could be indexed to layered birnessite MnO_2_ (JCPDS 13–0105), as shown in Supplementary Fig. 5. X-ray photoelectron spectroscopy (XPS) (Supplementary Fig. 6) shows a spin-energy separation of 4.81 eV for the Mn 3 s doublet in the PANI-intercalated MnO_2_ nanolayers, indicating ~4.0 charge state of Mn in the composite^39,40^.

### Electrochemical characterization

**Fig. 2:**
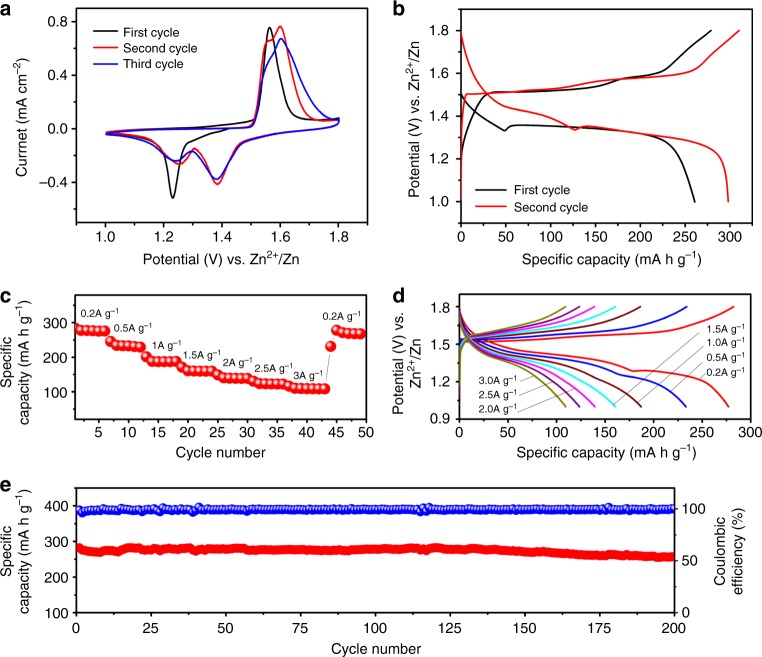
Electrochemical performance of polyaniline-intercalated MnO_2_ nanolayers. **a** Cyclic voltammetry curves of the coin-type cell (Zn/polyaniline (PANI)-intercalated MnO_2_) using 2 M ZnSO_4_ + 0.1 M MnSO_4_ aqueous electrolyte at 0.1 mV s^−1^. **b** Typical galvanostatic charge/discharge curves at 50 mA g^−1^ between 1.0 and 1.8 V of the cell. **c**, **d** Rate performance and charge/discharge profiles of the cell tested with the charge/discharge current densities varying from 200 to 3000 mA g^−1^. **e** Cycling performance in terms of specific capacity (red) and the corresponding coulombic efficiency (blue) at a current density of 200 mA g^−1^

The electrochemical profile of the PANI-intercalated MnO_2_ nanolayers composite is characterized by the typical coin-type cell, which is composed of a PANI-intercalated MnO_2_ composite cathode, a Zn foil anode, and an aqueous electrolyte (2 M ZnSO_4_ + 0.1 M MnSO_4_) adsorbed with a glass fiber separator. According to Liu’s report^23^, the presence of 0.1 M MnSO_4_ could inhibit the dissolution of Mn^2+^ (from Mn^3+^ disproportionation) into the electrolyte. Furthermore, the presence of Mn^2+^ can improve the Zn-platting/stripping efficiency (Supplementary Fig. 7). Figure 2a shows the cyclic voltammetry (CV) data for the PANI-intercalated MnO_2_ nanolayers composite. There is an obvious cathodic peak around 1.23 V during the first cathodic sweep, while the corresponding anode peak appears around 1.56 V during the anodic sweep. In the following cycles, the strengths of the redox peaks mentioned above gradually decrease; meanwhile, one new pair of redox peaks emerge around 1.38 and 1.60 V. The two-step charge storage should be attributed to the different insertion mechanism of H^+^ and/or Zn^2+^ during the discharge process, which will be further clarified in the mechanism investigation. The galvanostatic charge/discharge profile of the PANI-intercalated MnO_2_ nanolayers composite is shown in Fig. 2b, where the applied current density and the achieved capacity are calculated by mass loading of PANI-intercalated MnO_2_ nanolayers composite in the cathode (i.e., 2 mg cm^−2^ with an electrode area of 1 cm^−2^). When tested at the low current of 50 mA g^−1^ (0.16 C), the cell exhibits initial discharge capacity of 260 mA h g^−1^, involving a slope discharge profile from 1.5 to 1.33 V (~50 mA h g^−1^ capacity) and a consequent discharge platform about 1.36 V (~210 mA h g^−1^ capacity). In the subsequent cycle, the discharge capacity is increased to 298 mA h g^−1^, which is close to the theoretical capacity of 308 mA h g^−1^ (based on single electron transfer between Mn^4+^ and Mn^3+^). Figure 2c presents the rate performance tested at different current densities, and corresponding cycle profile is given in Fig. 2d. As shown in Fig. 2c, d, the cell exhibits a reversible discharge capacity of 280 mA h g^−1^ at the current density of 200 mA g^−1^, which is very close to that achieved at the low current density of 50 mA g^−1^. Even at the high current density of 3000 mA g^−1^, the cell still can deliver a capacity of 110 mA h g^−1^, which is to the best of our knowledge among the best rate performances reported to date in this field^23–25^. It should be noted that the two discharge plateaus evolve to a single one at the high rate, which should be attributable to H^+^ insertion that dominates the discharge process at high rate. This phenomenon can be explained by the faster H^+^ insertion than Zn^2+^ insertion, which will be confirmed by the later electrochemical impedance measurements. Cycle stability of the PANI-intercalated MnO_2_ composite was evaluated at the current densities of 200 and 2000 mA g^−1^. From Fig. 2e, it can be seen that PANI-intercalated MnO_2_ nanolayers composite delivers 280 mA h g^−1^ capacity for 200 cycles with coulombic efficiency around 100%, in which an ultra-high utilization of more than 90% (based on theoretical capacity of 308 mA h g^−1^ of MnO_2_) is obtained. To the best of our knowledge, it is the highest utilization that can be stable for 200 cycles in an aqueous zinc-ion battery (see Supplementary Table 1 for detailed information). The charge/discharge curves at different cycles are shown in Supplementary Fig. 8 to clarify the potential evolution during the cycling test, where a slight potential evolution over 200 cycles can be detected. When tested at the high current density of 2000 mA g^−1^, the PANI-intercalated MnO_2_ nanolayers composite present a stable discharge capacity of around 125 mA h g^−1^ (up to 40% utilization) over 5000 cycles (Supplementary Fig. 9). The stable cycle life of 5000 cycles with the utilization of 40% is superior to previous reports (see Supplementary Table 2 for detailed information). The superior performance is largely attributable to the reinforcement of the layered structure with intercalated PANI, which avoids phase transformation and collapse of the layered structure during repeated insertion/extraction of hydrated cations. Simultaneously, the presence of Mn^2+^ in the electrolyte also alleviates the Mn^2+^ dissolution-induced capacity fading^23,24^.

To further demonstrate the function of the PANI-reinforced layered structure, the cycling performance of the PANI-intercalated MnO_2_ nanolayers composite was also investigated using the ZnSO_4_ electrolyte without Mn^2+^ (Supplementary Fig. 10), and the corresponding result was compared with previous reports about MnO_2_ cycled in the electrolyte without Mn^2+^ (see Supplementary Table 3). On the other hand, it should be noted that the high cycle performance shown in Fig. 2e or Supplementary Fig. 9 is achieved by using excess Zn-anode (see Method section). The purpose is to exclude the effect of Zn-anode fading, which is similar with previous reports about MnO_2_ cathode^22–36^. SEM images of the cycled Zn electrode and PANI-intercalated MnO_2_ electrode are given in Supplementary Fig. 11 and Supplementary Fig. 12, respectively. In practical application, the issue of Zn-anode is another obstacle for Zn-ion batteries. The modification of an electrode developed by La Mantia et al^41^. and the electrolyte optimization reported by Chen’s group^42^ may be the potential solutions to improve the stability of Zn-anode.

### Reaction mechanism

**Fig. 3:**
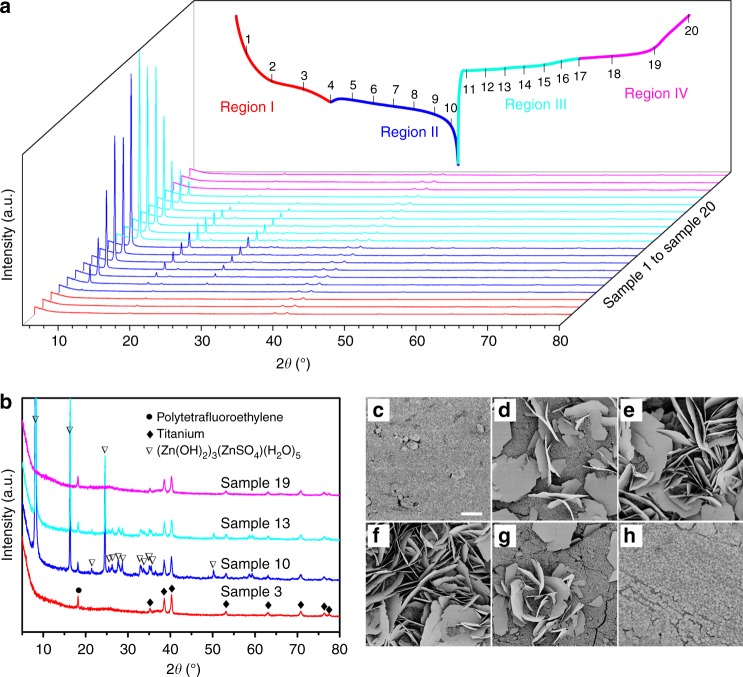
Structure evolution of polyaniline-intercalated MnO_2_ electrode during cycling. **a** Evolution of ex situ X-ray powder diffraction (XRD) patterns during the charge/discharge process (the vertical bars denoted with consecutive numbers indicate the locations where the XRD patterns were recorded). **b** Selected ex situ XRD patterns from **a** (corresponding to the XRD patterns denoted with 3, 10, 13, and 19 in **a**), which represent the typical XRD pattern in each corresponding charge/discharge region. **c**–**h** Scanning electron microscopy (SEM) images for morphologic evolution of electrode during cycling (the SEM images were taken at the locations indicated by vertical bars 3, 5, 10, 12, 16, and 20 in **a**, respectively). Scale bars, **c**–**h** 10 μm, respectively (the magnification is the same for images **c**–**h**)

Until now, two reaction mechanisms for a manganese dioxide cathode, involving Zn^2+^ and H^+^ insertion/extraction, respectively, have been reported^23–25,31^. Due to various crystallographic polymorphs of manganese dioxide, the reaction mechanism during cycling in neutral aqueous electrolytes remains a topic of discussion. Here the insertion mechanism was investigated to better understand the electrochemical reaction during cycling. Ex situ XRD analysis of the PANI-intercalated MnO_2_ electrode in 2 M ZnSO_4_ + 0.1 M MnSO_4_ electrolyte was conducted during the charge/discharge cycle within the potential window of 1.0–1.8 V at a current density of 50 mA g^−1^ (Fig. 3a and Supplementary Fig. 13). During the first discharge platform (Region I, red color), only two sets of peaks (related to polytetrafluoroethylene (PTFE) binder at 18° and Ti current collector at 38.4° and 40.2°) could be clearly observed, and there is no obvious variation throughout Region I. However, from the beginning of the second discharge platform (Region II, blue color), some new peaks arise, including a very strong peak at 8.1° and obvious peaks at 16.2° and 24.4° 2*θ*. During the subsequence charge process, the strength of arisen peaks decreases gradually (Region III, cyan color), and finally recover to the original pattern (Region IV, pink color) which is as same as Region I, indicating a good reversibility of electrode reaction. In order to analyze the evolution more clearly, several selected XRD patterns from Fig. 3a were presented in Fig. 3b. The emerging peaks (including strong peaks at 8.1°, 16.2°, and 24.4° 2*θ* and other subtle peaks highlighted with inverted triangles) are indexed to (Zn(OH)_2_)_3_(ZnSO_4_)(H_2_O)_5_ (zinc hydroxide sulfate hydrate, JCPDS: 78–0246)^23^. The formation of zinc hydroxide sulfate is in consistence with Liu et al.’s report^23^. With the consumption of H^+^ in the electrolyte, the increasing amount of OH^−^ leads to the formation of zinc hydroxide sulfate hydrate. SEM is further conducted to monitor the morphologic evolution of the PANI-intercalated MnO_2_ electrode (Fig. 3c–h). For Region I, there is no obvious change on the electrode surface, but in the Region II, increasingly large flakes emerge with discharging and gradually vanish during subsequent charging. The highly reversible morphologic transformation during the charge/discharge process is well consistent with the evolution observed in XRD patterns. Energy-dispersive spectroscopy (EDS) analysis shows that the flake-like product contains abundant Zn and S, but no evident Mn, which supports that the large flakes are zinc hydroxide sulfate hydrate (Supplementary Fig. 14), as indexed in the XRD patterns.

**Fig. 4:**
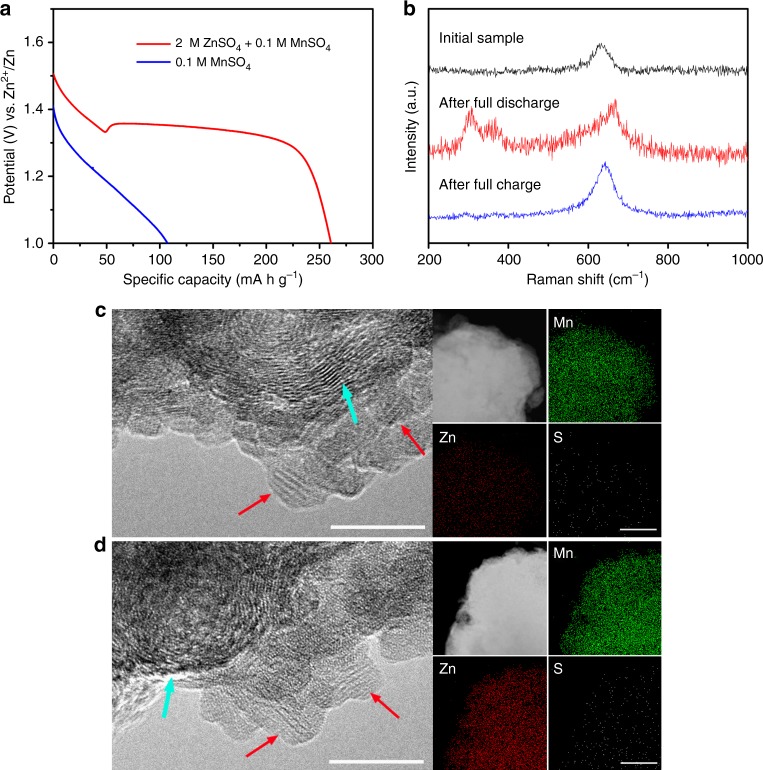
Characterization of sequential insertion of H^+^ and Zn^2+^ during two discharge platforms. **a** The discharge profile of polyaniline (PANI)-intercalated MnO_2_ electrode at current density of 50 mA g^−1^ in different electrolytes (red curve: 2 M ZnSO_4_ + 0.1 M MnSO_4_, blue curve: 0.1 M MnSO_4_). **b** Raman spectra of PANI-intercalated MnO_2_ electrode after full discharge and full charge. **c** High-resolution transmission electron microscopy (HR-TEM) image of the testing electrode after the first discharge platform and the corresponding scanning transmission electron microscopy–energy dispersive spectroscopy (STEM–EDS) mappings for elements like Mn, Zn, and S. **d** HR-TEM image of the testing electrode after the second discharge platform and the corresponding STEM–EDS mappings for elements like Mn, Zn, and S. Red arrows indicate the PANI-intercalated MnO_2_ nanolayers and cyan arrows indicate the acetylene black in electrode. Scale bars, **c**, **d** 10 nm for TEM images and 100 nm for STEM–EDS mapping images

As demonstrated by the above observation, the electrochemical reaction of the PANI-intercalated MnO_2_ nanolayers composite definitely involved H^+^ insertion, which supports the conclusion by Liu et al^23^. However, it does not preclude Zn^2+^ insertion during the discharge process. We presume that besides H^+^ insertion, Zn^2+^ insertion plays an important role in the discharge process because the two discharge platforms cannot be satisfactorily explained by only H^+^ insertion. Therefore, further investigation was performed to clarify this point. Figure 4a shows the discharge curves of the PANI-intercalated MnO_2_ nanolayers composite in different electrolytes (red curve: 2 M ZnSO_4_ + 0.1 M MnSO_4_, blue curve: 0.1 M MnSO_4_). As we know, PANI-intercalated MnO_2_ exhibits two discharge platforms in 2 M ZnSO_4_ + 0.1 M MnSO_4_ electrolyte (red curve). However, when Zn^2+^ was eliminated, a single-slope discharge profile was observed for the 0.1 M MnSO_4_ electrolyte (blue curve). From this result, we preliminarily conclude that the second discharge platform is related to Zn^2+^ insertion. Raman spectra (Fig. 4b) are used to further characterize Zn^2+^ insertion/extraction during charge/discharge states. A band of around 650 cm^−1^ can be observed throughout the whole charge/discharge process, which is attributed to the symmetric stretching vibration (Mn–O) of the MnO_6_ groups^43,44^. In addition, a pair of peaks between 300 and 400 cm^−1^ that are derived from Zn−O vibrations^45,46^ arise after discharge to 1 V and then vanish after consequent charging. This reversible Zn−O band demonstrates the insertion/extraction of Zn^2+^ in the PANI-intercalated MnO_2_ electrode. The conclusion is supported by the observation of Zn on the electrode surface with scanning electron microscopy–energy dispersive X-ray spectroscopy (SEM–EDX) analysis after discharge (Supplementary Fig. 15). Moreover, the kinetic behavior during the first and second discharge platform was investigated with electrochemical impedance spectroscopy (EIS, Supplementary Fig. 16), in which the calculated diffusion coefficient in the first discharge platform (5.84 × 10^−12^ cm^2^ s^−1^) is much higher than that in the second discharge platform (7.35 × 10^−14^ cm^2^ s^−1^), indicating different insertion ions during the two different discharge platforms.

HR-TEM was further employed to gain insight into the structure evolution during the two-stage discharge process. As seen in Fig. 4c, after the first discharge platform, the layered structure with large lattice spacing (see red arrows) is maintained (the cyan arrow indicates the acetylene black in the electrode), and the corresponding scanning transmission electron microscopy–energy dispersive spectroscopy (STEM–EDS) mapping reveals abundant Mn, trace amounts of Zn, and negligible S in the discharge products, indicating H^+^ insertion in the initial discharge stage. The layered structure was also preserved well after the second discharge platform (Fig. 4d), but unlike the first discharge platform, zinc is abundant with homogeneous distribution in the PANI-intercalated MnO_2_ nanolayers according to STEM–EDS mapping, confirming Zn^2+^ insertion into the PANI-intercalated MnO_2_ nanolayers. Notably, the layered structure was preserved after the long cycle test (Supplementary Fig. 17), which strongly demonstrates the high stability of PANI-intercalated MnO_2_ nanolayers. On the contrary, other MnO_2_ crystallographic polymorphs suffer severe phase transformation, as reported by previous researchers^24,31,32^.

**Fig. 5:**
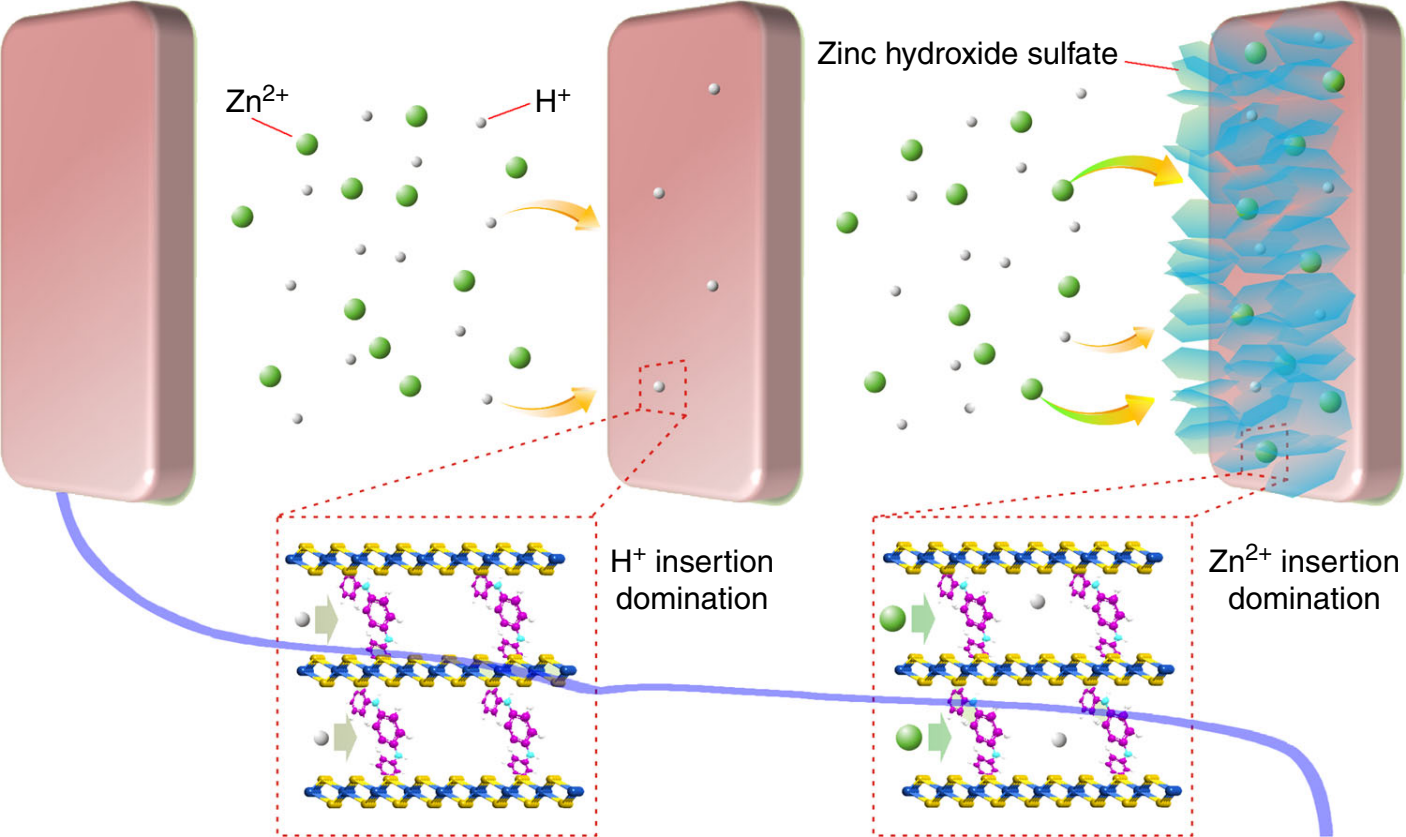
Diagram showing the sequential insertion of H^+^ and Zn^2+^. During the first discharge platform, H^+^ insertion into polyaniline (PANI)-intercalated MnO_2_ nanolayers dominates the electrode reaction, which gradually decreases H^+^ concentration around the electrode. With a sustained decrease of H^+^, Zn^2+^ insertion dominates the electrochemical reaction, raising the second discharge platform; meanwhile, the sustained decrease of H^+^ concentration leads to the formation of zinc hydroxide sulfate on the electrode surface

Based on the above analysis, we propose a co-insertion mechanism of H^+^ and Zn^2+^ in PANI-intercalated MnO_2_ nanolayers with a self-regulating function in the electrolyte (Fig. 5). In the first stage of discharge, H^+^ initially inserts into PANI-intercalated MnO_2_ nanolayers, leading to a gradual decrease of H^+^ concentration in the vicinity of the electrode. During the first discharge platform, the OH^−^ concentration is not high enough to form zinc hydroxide sulfate. With sustained decrease of H^+^ concentration, the second discharge platform arises, which is caused by a Zn^2+^ insertion reaction; meanwhile, the amount of zinc hydroxide sulfate formed on the electrode surface increases. Along with the Zn^2+^ insertion, H^+^ insertion is ongoing in the second discharge platform, leading to the increased formation of flake-like zinc hydroxide sulfate. Note that this “self-regulation function” consumes superfluous OH^−^, which is beneficial for high cycle stability. On recharge, the released H^+^ can lead to the dissolution of the zinc hydroxide sulfate.

## Discussion

In summary, PANI-intercalated MnO_2_ nanolayers were prepared and investigated as the cathode material for a rechargeable Zn–MnO_2_ battery using a mild aqueous electrolyte. With the typical nanosize, expanded interlayer space, uniform meso-structure and polymer-reinforced layered structure, the PANI-intercalated MnO_2_ nanolayers show a promising rate performance and an excellent cycling stability at high charge/discharge depth that is superior to previous reports. It is demonstrated that the PANI-reinforced layered structure combined with the nanoparticle-sized (~10 nm) MnO_2_ can efficiently eliminate the hydrated H^+^/Zn^2+^-insertion-induced phase transformation and the subsequent structure collapse, which is of vital significance to obtaining long cycle life along with high capacity utilization. Furthermore, the hydrated H^+^/Zn^2+^ co-insertion process in the layered MnO_2_ was investigated in detail, and a self-regulating mechanism of electrolyte-involved generation/dissolution of flake-like zinc hydroxide sulfate was clarified. These achievements cast light on the design of more advanced MnO_2_ cathode materials for rechargeable Zn–MnO_2_ batteries using mild aqueous electrolytes.

## Methods

### Material preparation

In a typical synthesis, aniline monomer (9 mL, Aldrich) was dissolved in CCl_4_ organic phase (450 mL, Aldrich) and potassium permanganate (0.45 g, Aldrich) was dissolved in distilled water (450 mL, pH 7). The solution was mixed to obtain an aqueous/organic stratification system with a clear interface. The reaction system was kept at 5 °C for 24 h. The chemical oxidation polymerization of aniline and reduction of potassium permanganate occurred simultaneously at the aqueous/organic interface, which is similar to our previous report^38^. By continuous diffusion of aniline from the organic phase to the aqueous phase, layer-by-layer self-assembly of layered manganese dioxide and polymer was established, and the final products were obtained after centrifugation and freeze drying.

### Characterization

Powder XRD patterns were collected on a X-ray diffractometer (Bruker D8 Advance, Germany) with Cu Kα radiation (*λ* = 0.15406 nm). SEM images and EDX mapping were obtained on Field-emission JEOL JSM-6390 microscope. TEM and EDS mapping were performed on JEOL JEM-2010 microscope. XPS was tested on a Thermo Escalab 250 equipped with a hemispherical analyzer. Raman spectra were obtained on RENISHAW inVia Raman Microscope using 633 nm excitation. T.G. was measured by a STA209 PC (NETZSCH, Germany) analyzer with an O_2_ flow. Fourier transform infrared spectroscopy (FT-IR) spectrum was recorded with a NICOLET 6700 FT-IR Spectrometer using KBr pellets.

### Electrochemical measurements

Electrochemical measurements were performed with CR2016 coin-type cells. The full cells were assembled using the PANI-intercalated MnO_2_ composite as the cathode, a zinc metal foil as the anode, a glass fiber as separator, and aqueous 2 M ZnSO_4_ with 0.1 M MnSO_4_ as electrolyte. The working electrode was fabricated by compressing a mixture of the active materials of PANI-intercalated MnO_2_ composite, the conductive material (acetylene black, AB), and the binder (polytetrafluoroethylene, PTFE) in a weight ratio of active materials/AB/PTFE = 80:10:10 onto a Ti grid at 20 MPa. The areal loading density of PANI-intercalated MnO_2_ is 2.0 mg cm^–2^, while the counter electrode (Zinc metal foil) is 20 mg cm^–2^ with the purpose of excluding the effect of Zn-anode fading. Galvanostatic charge/discharge performances were conducted on a battery test system (Neware BTS 4000). Cyclic voltammetry (0.1 mV s^–1^) and electrochemical impedance spectroscopy (an AC voltage of 5 mV amplitude) measurements were carried out using an AUTOLAB electrochemical work station (PGSTAT 302N). In order to avoid the conglutination between separator and electrode, the electrochemical tests for SEM, TEM, Raman, and XRD analysis were conducted with a simulated battery composed of a working electrode, counter electrode and electrolyte, but no separator.

### Data availability

The authors declare that all the relevant data are available within the paper and its Supplementary Information file or from the corresponding author on reasonable request.

## Electronic supplementary material

Supplementary Information
