# Supplementary material for: Polyaniline-intercalated manganese dioxide nanolayers as a high-performance cathode material for an aqueous zinc-ion battery
Source: Nat Commun. 2018 Jul 25;9:2906. doi: 10.1038/s41467-018-04949-4 (PMC6060179; doi:10.1038/s41467-018-04949-4)
Supplement: Supplementary file 1 — Supplementary Information [file 41467_2018_4949_MOESM1_ESM.pdf]

Supplementary Information

Polyaniline-intercalated manganese dioxide nanolayers as high performance cathode material for aqueous zinc-ion battery

Huang, *et al.*

Supplementary Figures

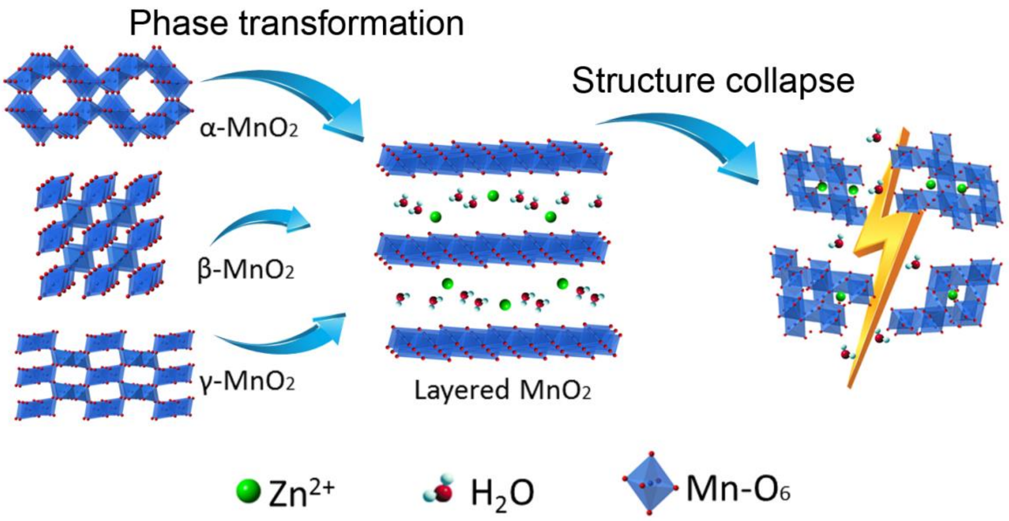

Supplementary Figure 1 | Structural evolution of manganese dioxide (MnO_2) during the charge/discharge. Reviewing the literature of zinc ion battery, most of cathode materials experience a phase transformation from initial structure to layered structure, and consequent structure collapse of layered structure arise during cycling.

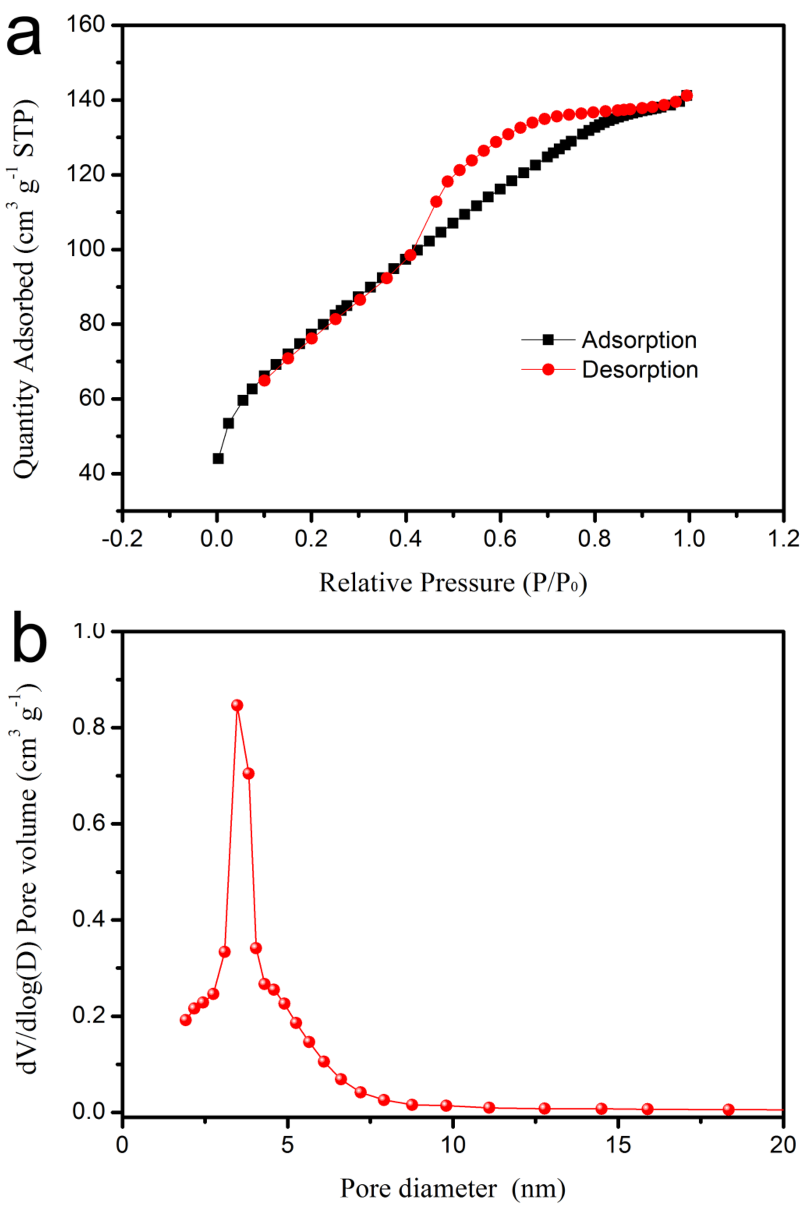

Supplementary Figure 2 | BET analysis of polyaniline (PANI)-intercalated MnO_2 nanolayers. (a) The nitrogen adsorption-desorption isotherms and **(b)** the corresponding pore size distributions of PANI-intercalated MnO_2 nanolayers. The obvious hysteresis loop in the relative pressure range of 0.4-0.8 indicates the typical mesoporous structure of PANI-intercalated MnO_2 nanolayers. And the pore size distribution of PANI-intercalated MnO_2 nanolayers is mainly centered around 4 nm. Furthermore, the surface area of PANI-intercalated MnO_2 nanolayers is 277 m^2 g^{-1}.

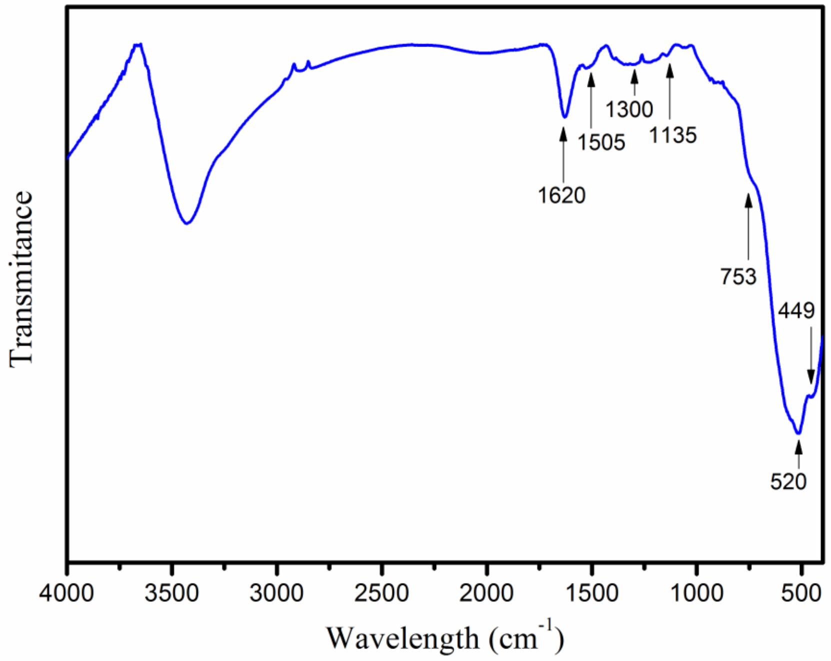

Supplementary Figure 3 | FTIR analysis of PANI-intercalated MnO_2 nanolayers. The band around 1505 cm^{-1} is corresponded to C=C stretching of quinoid; and the bands range 1200–1400 cm^{-1} are assigned to C–N stretching of aromatic amine and N-H bending mode; the band at 1135 cm^{-1} is related to the degree of electron delocalization in PANI and stretching of N=Q=N in quinoid (Q) ring. In addition, other characteristic absorption bands in the range of 400-800 cm^{-1} are assigned to Mn-O stretching vibrations of MnO_6 octahedra (refs.4 and 5).

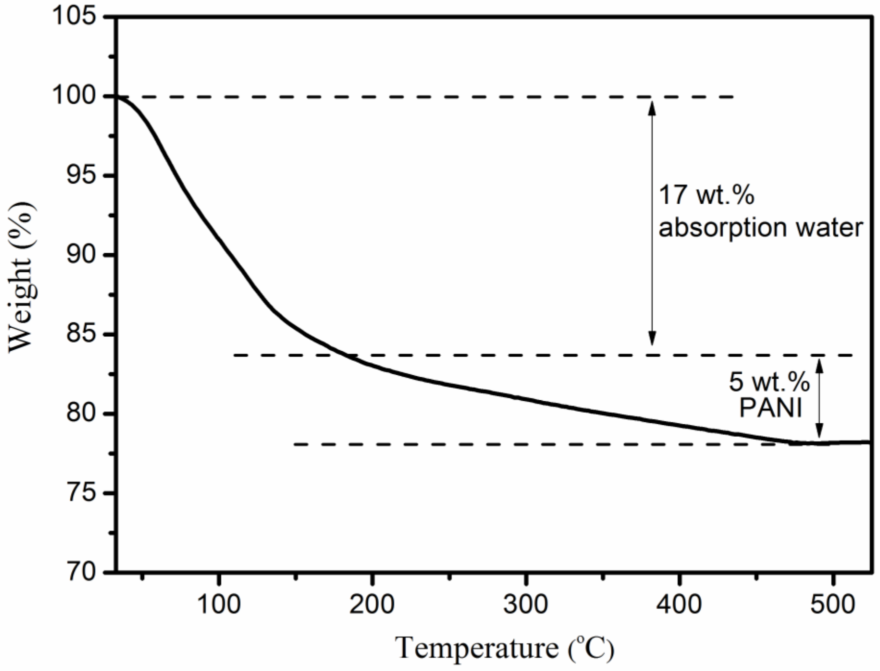

Supplementary Figure 4 | TG analysis of PANI-intercalated MnO_2 nanolayers. The first weight loss (~17%) below 200 °C is attributed to the loss of adsorbed water and the second weight loss (~5%) between 200 °C and 500 °C was due to the loss of PANI.

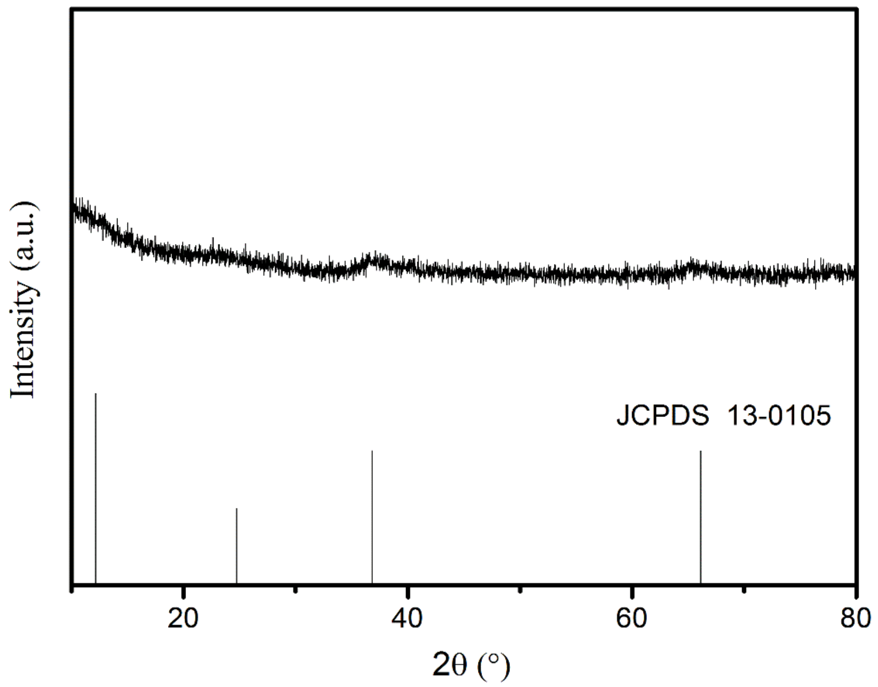

Supplementary Figure 5 | XRD pattern of PANI-intercalated MnO_2 nanolayers. The broadened diffraction peak with low intensity can be indexed to the layered birnessite MnO_2 (JCPDS 13-0105). The broad and weak features indicate the typical characteristic of nano-crystalline for PANI-intercalated MnO_2 nanolayers.

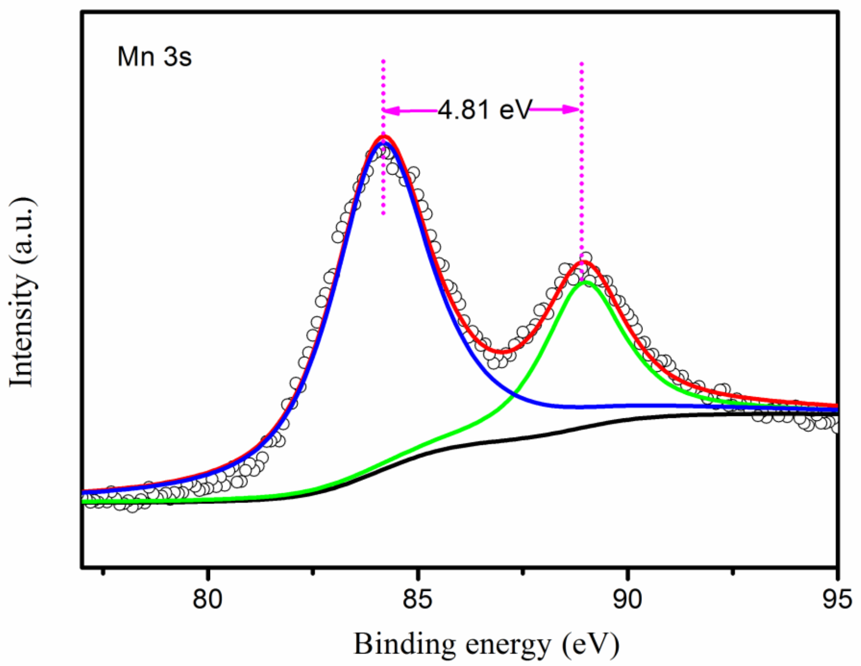

Supplementary Figure 6 | XPS spectra of Mn 3s doublet for PANI-intercalated MnO_2 nanolayers. The spin-energy separation of 4.81 eV for the Mn 3s doublet indicates ~4.0 charge state of Mn in the composite.

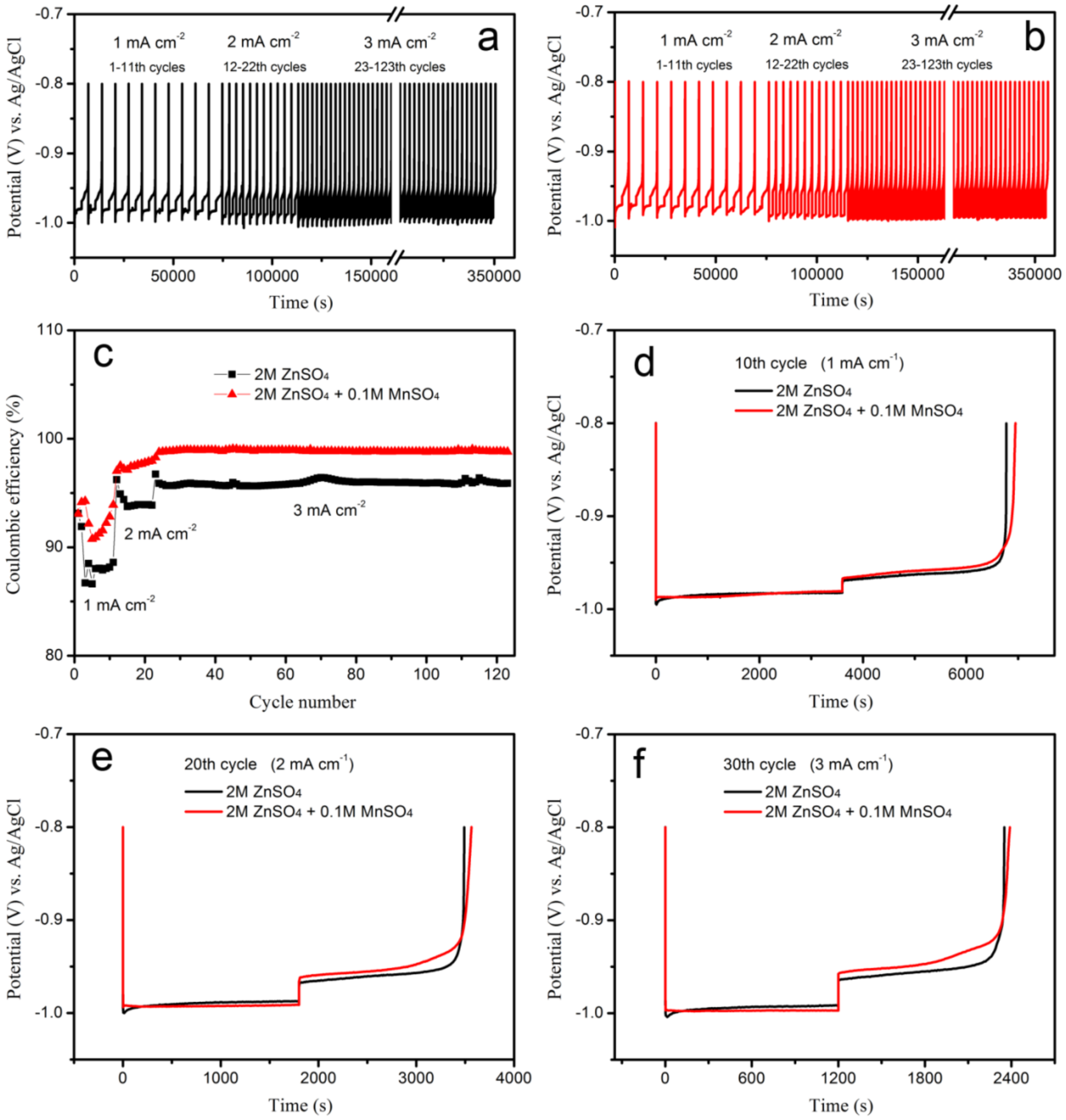

Supplementary Figure 7 | Platting/stripping profile of Zn in the electrolyte with or without Mn^{2+}. The platting/stripping tests were conducted at a fixed platting capacity of 1mAh cm^{-2} with an applied current of 1 mA cm^{-2}, 2 mA cm^{-2} and 3 mA cm^{-2}, respectively. Working electrode: Cu foil; Counter electrode: Zn foil; Reference electrode: Ag/AgCl electrode. **(a)** Zn-platting/stripping behaviors (potential *vs*. time) in 2M $ZnSO_4$ electrolyte and **(b)** in 2M $ZnSO_4$ + 0.1M $MnSO_4$ electrolyte. **(c)** Platting/stripping efficiency. Comparison of Zn-platting/stripping profile at **(d)**10th cycle (potential *vs*. time, 1 mA cm^{-2}), **(e)** 20th cycle Zn-platting/stripping profile (potential *vs*. time, 2 mA cm^{-2}) and **(f)** 30th cycle Zn-platting/stripping profile (potential *vs*. time, 3 mA cm^{-2}), respectively.

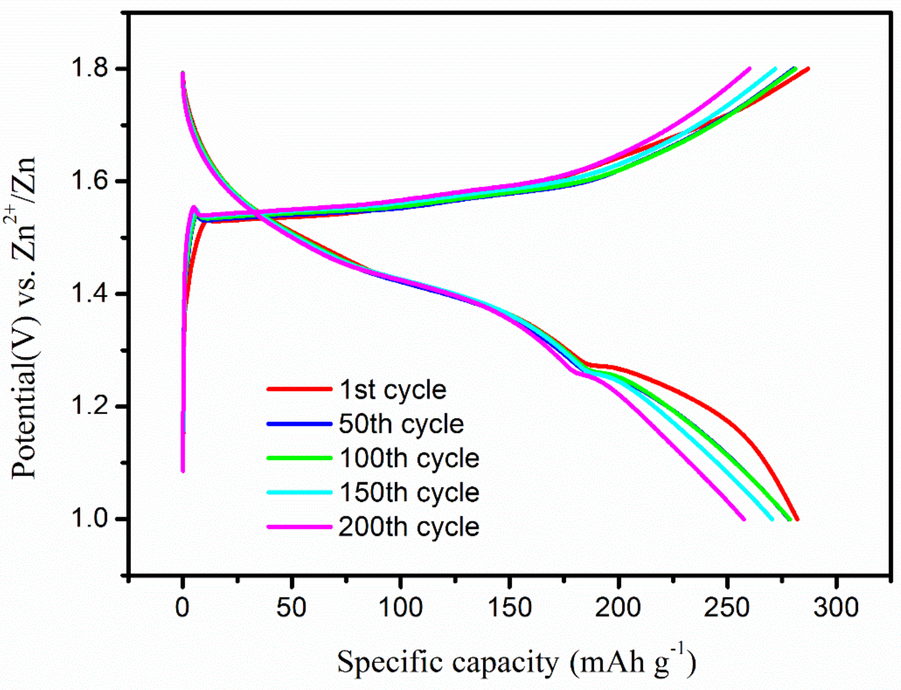

Supplementary Figure 8 | The evolution of the charge/discharge curves during cycling at current density of 200 mA g^{-1}.

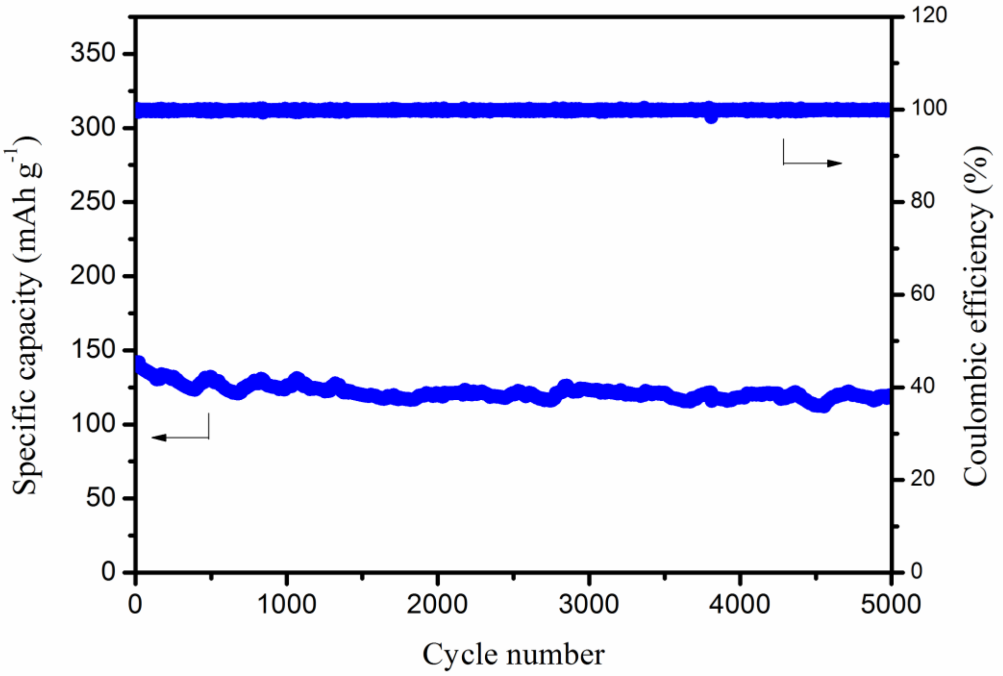

Supplementary Figure 9 | Long-cycle performance of PANI-intercalated MnO_2 nanolayers at a high current density of 2000 mA g^{-1}. Tested with a CR2016 coin-type cell, which is composed of a PANI-intercalated MnO_2 cathode, a Zn foil anode and the aqueous electrolyte (2 M $ZnSO_4$ + 0.1 M $MnSO_4$) adsorbed with a glass fiber separator.

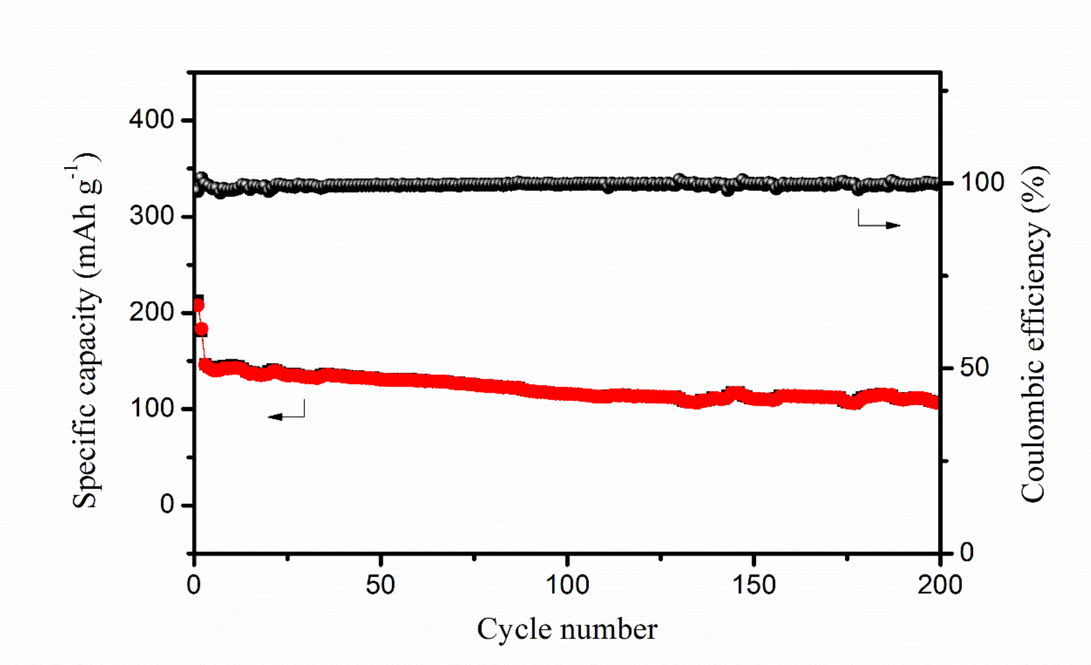

Supplementary Figure 10 | Cycle performance of the PANI-intercalated MnO_2 nanolayers using the 2M $ZnSO_4$ electrolyte without Mn^{2+} tested at an applied current density of 200 mA g^{-1}. The capacity fade is serious in the first few cycles (1st to 5th), which might mainly arising from the Mn^{2+} dissolution (The formation of Mn^{2+} is mainly attributable to the Mn^{3+} disproportionate). Then, the capacity fade becomes very slow on the subsequent cycles (5th to 200th), which is much superior to the continuous capacity fade reported in previous works about the MnO_2-cathode cycled in the electrolyte without Mn^{2+} (Supplementary Table 3). Note that due to the continuous capacity fade, there are no previous reports that can achieve 200 cycles while keeping >100 mAh g^{-1} discharge capacity in $ZnSO_4$ electrolyte. The results confirm that PANI in the interlayer can increase the cycle life of MnO_2 cathode. On the other hand, the achieved capacity is still lower than that achieved in the electrolyte containing Mn^{2+} (see Figure 2c in main-text). The capacity fade of the MnO_2 depends on both the Mn^{2+} dissolution and the phase-transition-induced gradual collapse of the tunnels or layers upon electrochemical cycling (Refs.1,2,6,7). For our case, the presence of Mn^{2+} in the electrolyte can efficiently alleviate the Mn^{2+} dissolution caused capacity fade, and the PANI-strengthened layered structure can efficiently alleviate the structure collapse induced capacity fade.

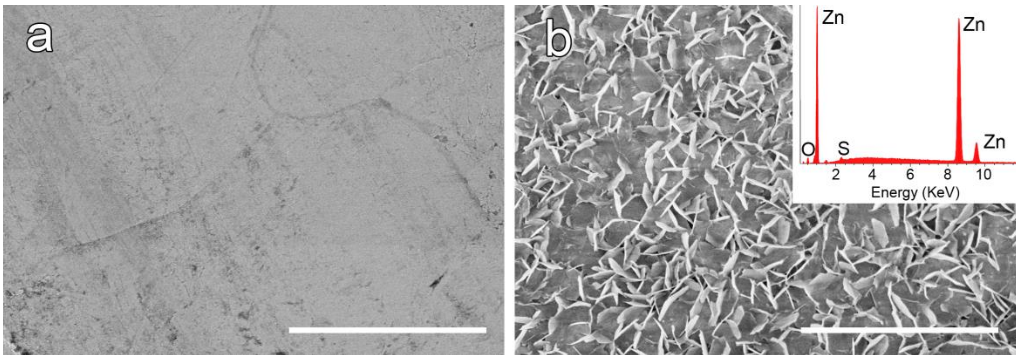

Supplementary Figure 11 | Surface morphology of **(a)** original Zn electrode and **(b)** cycled Zn electrode (inset: EDX spectrum of cycled Zn electrode). Scale bars, 5 μm, (**a, b**).

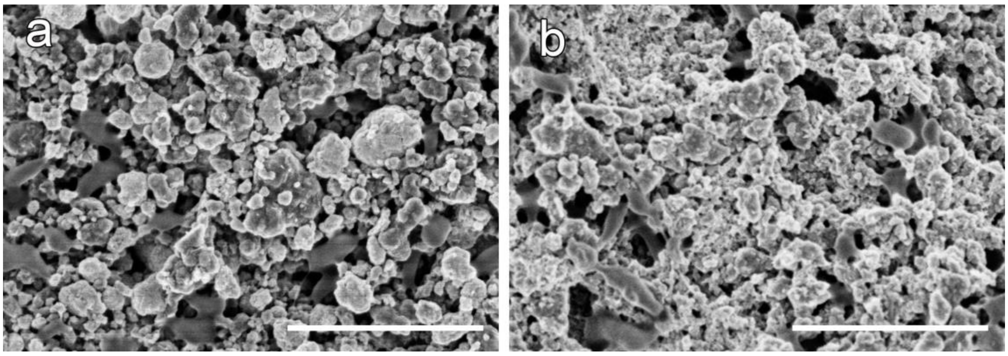

Supplementary Figure 12 | Surface morphology of **(a)** original PANI-intercalated MnO_2 electrode and **(b)** cycled PANI-intercalated MnO_2 electrode. Scale bars, 1 μm, (**a, b**).

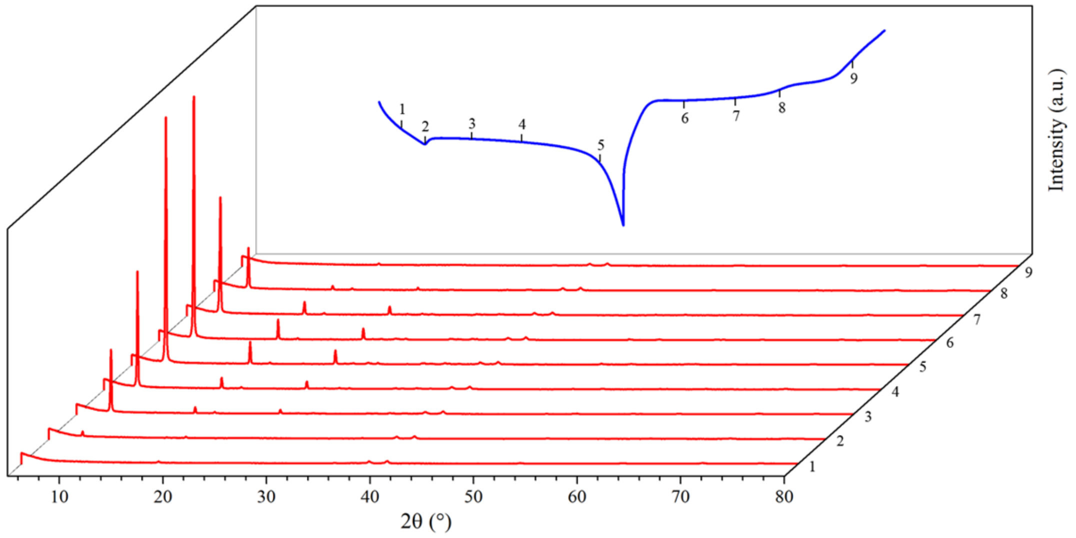

Supplementary Figure 13 | Structure evolution of PANI-intercalated MnO_2 electrode during the first charge/discharge cycle. There is the first charge/discharge profile on the top of the figure, the evolution of XRD pattern for the first cycle is almost same as that for the second cycle.

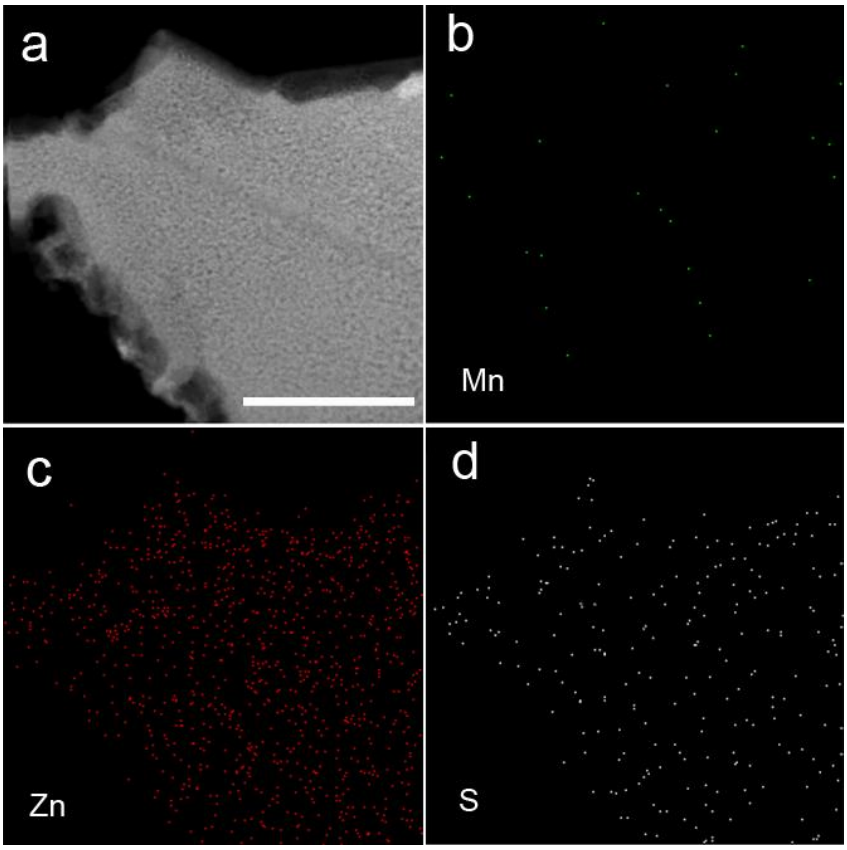

Supplementary Figure 14 | STEM image and according EDS mapping of flake-like product on electrode surface. The presence of Zn, S and the absence of Mn verify the fact that the ingredient of flake-like product is zinc hydroxide sulfate. Scale bars, 400 nm.

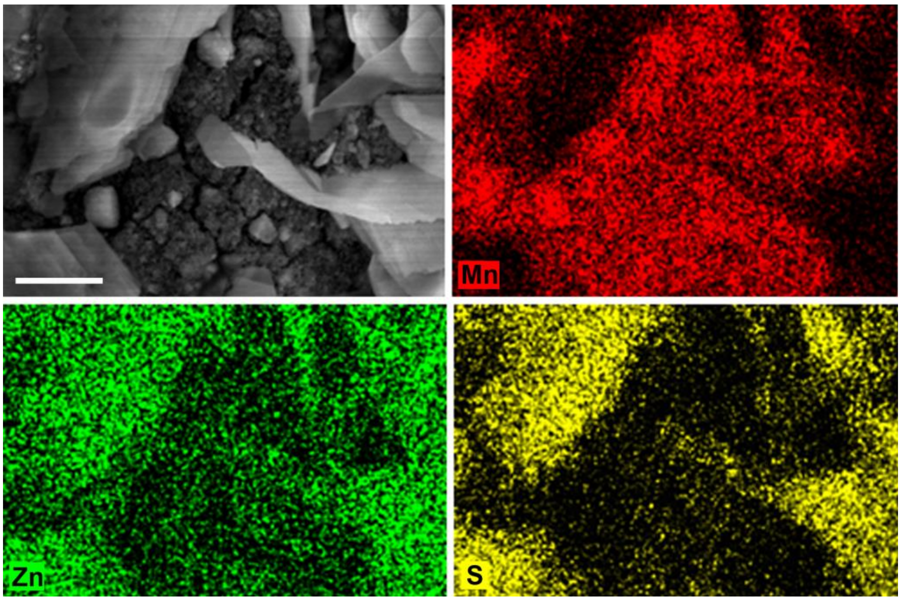

Supplementary Figure 15 | Element mapping on the surface of PANI-intercalated MnO_2 electrode during second discharge platform. It can be seen that Mn and S is mainly enriched in MnO_2 and flake zinc hydroxide sulfate, respectively. The presence of Zn and the absence of S on the exposed surface of PANI-intercalated MnO_2 electrode indicate the insertion of Zn into MnO_2. Although Zn could be observed in both MnO_2 and zinc hydroxide sulfate, the concentration of Zn in flake is higher than that in MnO_2, it is reasonable because the Zn content in zinc hydroxide sulfate is higher than that inserted into MnO_2. Scale bars, 40 μm.

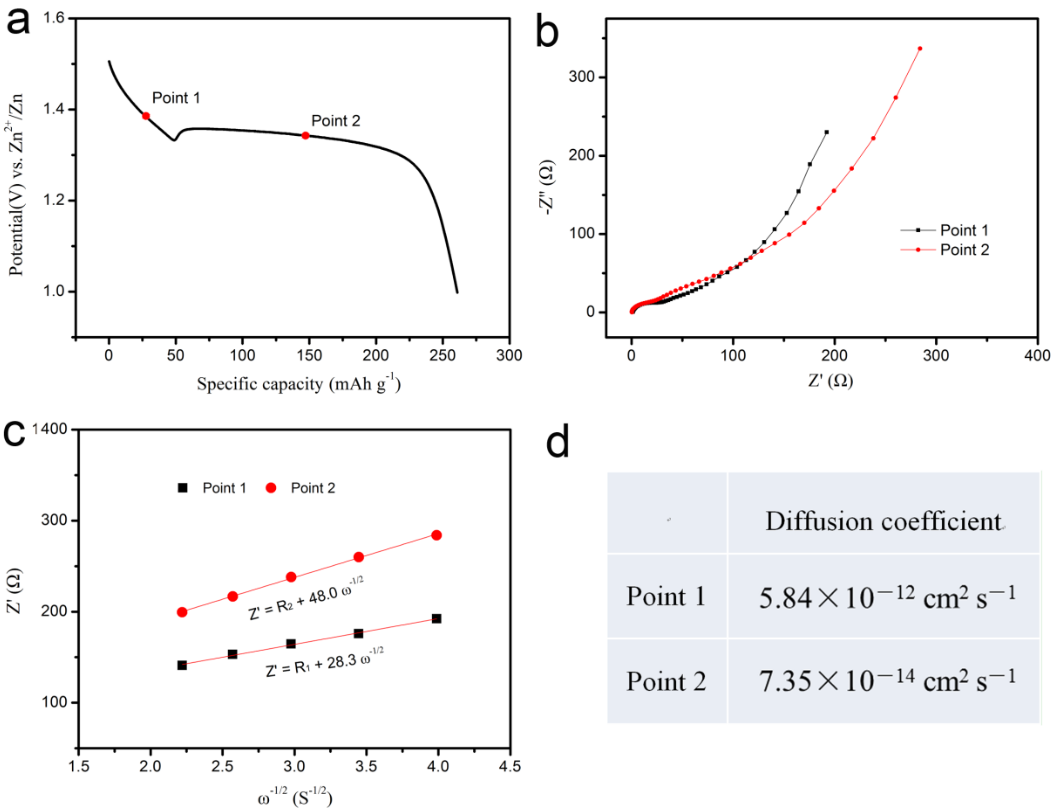

	Diffusion coefficient
Point 1	5.84×10^{-12} cm^2 s^{-1}
Point 2	7.35×10^{-14} cm^2 s^{-1}

Supplementary Figure 16 | Electrochemical impedance spectroscopy (EIS) analysis and the calculated diffusion coefficient. A T-type cell with three electrodes is employed to conduct the impedance measurements (Working electrode: PANI-intercalated MnO_2 nanolayers; Counter electrode: Zn foil; Reference electrode: Zn foil). **(a)** The points on the discharge curve indicate the position where EIS test is conducted; **(b)** Nyquist spectra measured at different discharge depth according to Point 1 and Point 2, respectively; **(c)** Z' *vs.* $\omega^{-1/2}$ plots in the low frequency region obtained from according EIS measurements; **(d)** The calculated diffusion coefficient at different discharge states. The diffusion coefficient on Point 2 is much lower than that for Point 1, considering the much larger size and electrostatic repulsion of Zn^{2+} than H^+, it is highly possible that the significant difference is caused by the different ion insertion.

The diffusion coefficient can be calculated based on following equation (refs.8-10):

$$D = \frac{R^2T^2}{2A^2n^4F^4C^2\sigma^2}$$

Where R is the gas constant, T is the experiment temperature, A is the surface area of the electrode, n is the number of the electrons per molecule attending the electronic transfer reaction, F is the Faraday constant, C is the concentration of insertion ion in MnO_2, and σ is the slope of the line Z' *vs.* $\omega^{-1/2}$ obtained from (c).

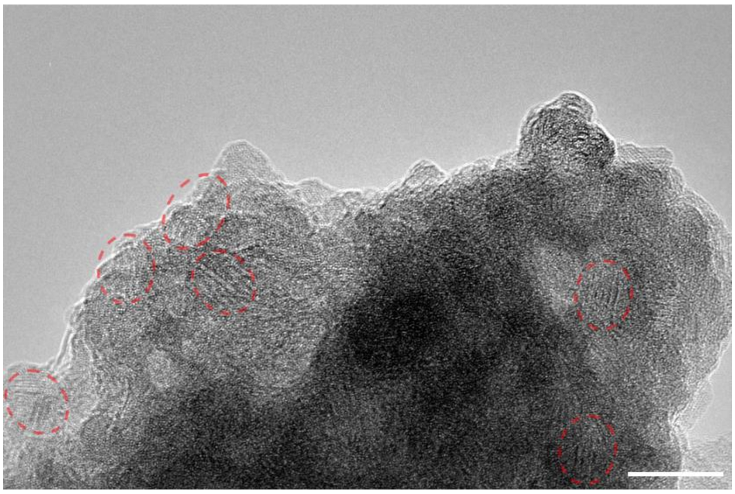

Supplementary Figure 17 | TEM image of PANI-intercalated MnO_2 nanolayers after cycling. The red cycles highlight the maintained layered structure of PANI-intercalated MnO_2 nanolayers. Scale bars, 10 nm.

Supplementary Note 1: It has been widely reported that various MnO_2 hosts (e.g. α, β and γ–phase, etc.) suffer the structure transformation during discharge/charge cycles in the Zn^{2+}-containing mild aqueous electrolyte, and a layered MnO_2 with interlaminar water molecules (i.e., buserite-phase with a much larger interlayer distance) is generally formed. The formation of layered structure with the larger interlayer distance arises from the dissolution of Mn^{2+} and the hydrated Zn^{2+} insertion (Supplementary Refs. 1, 2). For example, in the investigation about the insertion meshcinsm of Zn^{2+} into α-MnO_2 (Supplementary Ref. 1), Oh *et al.* demonstrated that Mn^{2+}-dissolution from the channel wall results in the formation of layered birnessite (that is a typical layer structure). Then, Oh *et al.* further demonstrated that the Zn^{2+}-inserted layered MnO_2 exhibits a much larger interlayer distance of 1.1 nm, and claimed that the larger interlayer distances should be attributable to the insertion of hydrated Zn^{2+} (Supplementary Ref. 2). Furthermore, they also pointed out that the high ionic charge of the central cation (i.e., Zn^{2+}) can be distributed by the partial charges formed by polarized intimate water molecules, which can facilitate diffusion of Zn^{2+}, as the electrostatic interactions arise between Zn^{2+} ions and the framework of the MnO_2 hosting material (Supplementary Ref. 2). On the other hand, very recently, the hydrated Zn^{2+} insertion into the layered V_2O_5 was also reported (Supplementary Ref. 3). For instance, Mai *et al.* demonstrated that the H_2O-solvated Zn^{2+} (i.e., the hydrated Zn^{2+}) possess largely reduced effective charge and thus reduced electrostatic interactions with the V_2O_5 framework, effectively promoting its diffusion (Supplementary Ref. 3).

Supplementary Tables

Supplementary Table 1. Comparison of utilization and cycle life of cathode materials for Zn-MnO_x battery with mild aqueous electrolyte at low discharge current.

References	Utilization	Cycle life (number of cycles)	Capacity (mAh g^{-1})	Current density (mA g^{-1})	Mass loading (mg cm^{-2})
6	75%	150	230	200	2
11	42%	100	130	150	--
12	84%	45	260	300	2
13	49%	300	150	400	0.6
14	49%	100	150	83	--
15	32%	50	100	50	2
16	45%	30	140	100	--
Our work	90%	200	280	200	2

Supplementary Table 2. Comparison of utilization and cycle life of cathode materials for Zn-MnO_x battery with mild aqueous electrolyte at high discharge current.

References	Utilization	Cycle Life (number of cycles)	Capacity (mAh g^{-1})	Current Density (mA g^{-1})	Mass loading (mg cm^{-2})
6	45%	2000	140	2000	2
11	32%	100	100	1800	--
12	29%	5000	90	1500	2
13	21%	10000	65	2000	0.6
16	24%	2000	75	2000	--
17	29%	500	90	500	2
Our work	40%	5000	125	2000	2

Supplementary Table 3. Comparison of the achieved cycle performance in previous reports about the MnO_2-cathode in the Zn-containing electrolyte without the presence of Mn^{2+}.

References	Achieved cycles	Initial cycle capacity (mAh g^{-1})	10th cycle capacity (mAh g^{-1})	End cycle capacity (mAh g^{-1})	Current density (mA g^{-1})
6	70	220	140	90 (70cycles)	200
11	100	165	160	105 (100cycles)	150
14	100	250	190	110 (100cycles)	83
15	50	110	90	90 (50cycles)	50
16	30	135	145	120 (30cycles)	100
Our work	200	210	140	110 (200cycles)	200

Supplementary References

1. Lee, B. et al. Electrochemically-induced reversible transition from the tunneled to layered polymorphs of manganese dioxide. *Sci. Rep.* **4**, 6066 (2014).
2. Lee, B. et al. Elucidating the intercalation mechanism of zinc ions into alpha-MnO_2 for rechargeable zinc batteries. *Chem. Commun.* **51**, 9265–9268 (2015).
3. Yan, M. et al. Water-Lubricated Intercalation in $V_2O_5{\cdot}nH_2O$ for High-Capacity and High-Rate Aqueous Rechargeable Zinc Batteries. *Adv. Mater.* **30**, 1703725 (2017).
4. Anu Prathap, M. U., Satpati, B. & Srivastava, R. Facile preparation of polyaniline/MnO_2 nanofibers and its electrochemical application in the simultaneous determination of catechol, hydroquinone, and resorcinol. *Sensors Actuat. B: Chem.* **186**, 67–77 (2013).
5. Wang, Y. G., Li, H. Q. & Xia, Y. Y. A Polyaniline-Intercalated Layered Manganese Oxide Nanocomposite Prepared by an Inorganic/Organic Interface Reaction and Its High Electrochemical Performance for Li Storage. *Adv. Mater.* **18**, 2619–2623 (2006).
6. Zhang, N. et al. Rechargeable aqueous zinc-manganese dioxide batteries with high energy and power densities. *Nat. Commun.* **8**, 405 (2017).
7. Alfaruqi, M. H. et al. Electrochemically Induced Structural Transformation in a γ-MnO_2 Cathode of a High Capacity Zinc-Ion Battery System. *Chem. Mater.* **27**, 3609–3620 (2015).
8. Liu, H. et al. Kinetic study on $LiFePO_4$/C nanocomposites synthesized by solid state technique. *J. Power Sources* **159**, 717–720 (2006).
9. Shaju, K. M., Subba Rao, G. V. & Chowdari, B. V. R. EIS and GITT studies on oxide cathodes, O2-$Li_{(2/3)+x}(Co_{0.15}Mn_{0.85})O_2$ (x = 0 and 1/3). *Electrochim. Acta* **48**, 2691–2703 (2003).
10. Liu, Y., Zhou, Y., Zhang, J., Zhang, S. & Ren, P. The relation between the structure and electroche mical performance of sodiated iron phosphate in sodium-ion batteries. *J. Power Sources* **314**, 1–9 (2016).
11. Xu, C., Li, B., Du, H. & Kang, F. Energetic Zinc Ion Chemistry: The Rechargeable Zinc Ion Battery. *Angew. Chem. Int. Edit.* **51**, 933–935 (2012).
12. Pan, H. L. et al. Reversible aqueous zinc/manganese oxide energy storage from conversion reactions. *Nat. Energy* **1**, 1639 (2016).
13. Sun, W. et al. Zn/MnO_2 Battery Chemistry With H^+ and Zn^{2+} Coinsertion. *J. Am. Chem. Soc.* **139**, 9775–9778 (2017).
14. Alfaruqi, M. H. et al. A layered δ-MnO_2 nanoflake cathode with high zinc-storage capacities for eco-friendly battery applications. *Electrochem. Commun.* **60**, 121–125 (2015).
15. Lee, J., Ju, J. B., Cho, W. I., Cho, B. W. & Oh, S. H. Todorokite-type MnO_2 as a zinc-ion intercalating material. *Electrochim. Acta* **112**, 138–143 (2013).
16. Jiang, B. et al. Manganese Sesquioxide as Cathode Material for Multivalent Zinc Ion Battery with High Capacity and Long Cycle Life. *Electrochim. Acta* **229**, 422–428 (2017).
17. Zhang, N. et al. Cation-Deficient Spinel $ZnMn_2O_4$ Cathode in $Zn(CF_3SO_3)_2$ Electrolyte for Rechargeable Aqueous Zn-Ion Battery. *J. Am. Chem. So*c. **138**, 12894–12901 (2016).
